# Shared Genetic Influences Between Attention-Deficit/Hyperactivity Disorder (ADHD) Traits in Children and Clinical ADHD

**DOI:** 10.1016/j.jaac.2015.01.010

**Published:** 2015-04

**Authors:** Evie Stergiakouli, Joanna Martin, Marian L. Hamshere, Kate Langley, David M. Evans, Beate St Pourcain, Nicholas J. Timpson, Michael J. Owen, Michael O'Donovan, Anita Thapar, George Davey Smith

**Affiliations:** a Medical Research Centre (MRC) Integrative Epidemiology Unit (IEU) at the University of Bristol, Bristol, UK; b MRC Centre for Neuropsychiatric Genetics and Genomics, Institute of Psychological Medicine and Clinical Neurosciences, Cardiff University School of Medicine, Cardiff, UK; c School of Psychology, College of Biomedical and Life Sciences, Cardiff University; d University of Queensland Diamantina Institute, Translational Research Institute, Brisbane, Queensland, Australia; e School of Oral and Dental Sciences, University of Bristol, and the School of Experimental Psychology, University of Bristol

**Keywords:** attention-deficit/hyperactivity disorder (ADHD), polygenic risk scores, Avon Longitudinal Study of Parents and Children (ALSPAC), common variants, genetics

## Abstract

**Objective:**

Twin studies and genome-wide complex trait analysis (GCTA) are not in agreement regarding heritability estimates for behavioral traits in children from the general population. This has sparked a debate on the possible difference in genetic architecture between behavioral traits and psychiatric disorders. In this study, we test whether polygenic risk scores associated with variation in attention-deficit/hyperactivity disorder (ADHD) trait levels in children from the general population predict ADHD diagnostic status and severity in an independent clinical sample.

**Method:**

Single nucleotide polymorphisms (SNPs) with *p* < .5 from a genome-wide association study of ADHD traits in 4,546 children (mean age, 7 years 7 months) from the Avon Longitudinal Study of Parents and Children (ALSPAC; general population sample) were selected to calculate polygenic risk scores in 508 children with an ADHD diagnosis (independent clinical sample) and 5,081 control participants. Polygenic scores were tested for association with case-control status and severity of disorder in the clinical sample.

**Results:**

Increased polygenic score for ADHD traits predicted ADHD case-control status (odds ratio = 1.17 [95% CI = 1.08–1.28], *p* = .0003), higher ADHD symptom severity (β = 0.29 [95% CI = 0.04–0.54], *p* = 0.02), and symptom domain severity in the clinical sample.

**Conclusion:**

This study highlights the relevance of additive genetic variance in ADHD, and provides evidence that shared genetic factors contribute to both behavioral traits in the general population and psychiatric disorders at least in the case of ADHD.

Traditional behavioral genetic studies have shown that psychiatric disorders, whether defined categorically as diagnoses or viewed as trait measures, are moderately to highly heritable.^1^ Findings from these family, twin, and adoption studies also suggest that many childhood psychiatric disorders, such as attention-deficit/hyperactivity disorder (ADHD),^2,3^ autism,^4^ and depression^5^ can be viewed as extremes of dimensional attributes present in the general population. Epidemiological studies similarly suggest that many forms of psychopathology are underpinned by dimensions because there appear to be no thresholds beyond which links with risk factors and adverse outcomes appear.^3,6^ This observation also applies to many physical disorders such as obesity and cardiovascular disease.^7,8^ However, it has been much more difficult to demonstrate links between childhood behavioral traits and psychiatric disorder at the level of molecular genetics.^9^ Moreover, there is no evidence as yet to suggest whether the same risk alleles that contribute to behavioral traits in the general population also confer risk of psychiatric disorder.

Additive effects of common gene variants (single nucleotide polymorphisms [SNPs]), when jointly considered in terms of estimated heritability or polygenic risk scores, have been found to contribute to psychiatric disorders: specifically ADHD, autism, depression, bipolar disorder, and schizophrenia.^10^ A recent analysis of the largest international dataset of these disorders found that variation tagged by common SNPs explained 17% to 29% of variance in liability.^10^ In contrast, common variants did not contribute to the heritability of behavioral traits in children at age 12 from the general population using genome-wide complex trait analysis (GCTA), although this was not the same for cognitive traits.^11^ The difference in results between diagnoses and dimensions could be methodological, perhaps due to the fact that case-control studies analyze hypothetical constructs of continuous liabilities. However, it has been suggested that perhaps the genetic architecture of psychiatric disorders is different to that of behavioral traits.^12^ Given that this is not what is suggested by epidemiological studies of behavioral traits in children during early childhood or late adolescence,^4^ this hypothesis needs to be tested directly.

Previous research suggests that polygenic risk scores can distinguish children with clinically diagnosed ADHD from control participants.^13^ There is also some evidence that for ADHD, at least, polygenic risk scores associated with categorical diagnosis contribute to trait variation in individuals from the general population without the disorder. We previously found that polygenic risk scores associated with ADHD diagnosis predicted ADHD trait levels in children from the general population.^14^

In the present study, we derived polygenic risk scores from a discovery genome-wide association study (GWAS) of ADHD traits in a general population sample to test whether common genetic risk alleles associated with variation in ADHD trait levels predict ADHD diagnostic status and also the severity of disorder in an independent clinical case-control ADHD sample.

## Method

The polygenic risk score method described by the International Schizophrenia Consortium (ISC) was used in this analysis.^15^ A discovery quantitative GWAS of ADHD traits in the Avon Longitudinal Study of Parents and Children (ALSPAC) was used to identify risk alleles associated with higher levels of ADHD traits. Polygenic risk scores based on these population risk alleles were then calculated in the target patient sample, which was a published GWAS of British children with ADHD and population controls.^16^

### Avon Longitudinal Study of Parents and Children (ALSPAC)—Discovery Sample

ALSPAC, which was used as the discovery sample for this study, is a prospective birth cohort that recruited pregnant women with expected delivery dates between April 1991 and December 1992 from Bristol, United Kingdom. A total of 14,541 pregnant women were initially enrolled, with 14,062 children born. Detailed information on health and development of children and their parents was collected from regular clinic visits and completion of questionnaires. A detailed description of the cohort has been published previously.^17,18^ The study website contains details of all data that are available through a fully searchable data dictionary (http://www.bris.ac.uk/alspac/researchers/data-access/data-dictionary/). Ethical approval was obtained from the ALSPAC Law and Ethics Committee and the local ethics committees.

### ALSPAC GWAS Data

A total of 9,912 research participants were genotyped using the Illumina HumanHap550 quad genome-wide SNP genotyping platform by the Wellcome Trust Sanger Institute (Cambridge, UK) and the Laboratory Corporation of America (Burlington, NC) using support from 23andMe. Details on the quality control (QC) procedure have been published previously.^19^ The resulting data set consisted of 8,365 participants and 500,527 SNPs available for analysis. Association analyses were performed under an additive model using PLINK,^20^ with sex included in the analysis as a covariate.

### Phenotypic Measures

ADHD traits were assessed in ALSPAC when the participants were 7 years 7 months of age using the parent-completed Development and Well-Being Assessment (DAWBA).^21^ The time point was chosen to be as close as possible to the mean age of children with ADHD in the target sample while at the same time maximizing sample size. For each ADHD item, parents marked boxes to say whether their child showed the behavior; these were coded 0 for “no,” 1 for “a little more than others,” and 2 for “a lot more than others.” A total ADHD trait score was calculated by summing these responses to give a possible range of 0 to 36. Scores on measures with less than 30% missing items were mean imputed. IQ was assessed using a short form of the Wechsler Intelligence Scale for Children (WISC)–III assessment with alternate items from all 10 subtests administered.^22^ The GWAS was performed after excluding children with a diagnosis of autism spectrum disorder (ASD), those with Full Scale IQ less than 70, and those for whom there were no data available on ASD diagnosis or IQ, to follow the exclusion criteria that were used in the target sample.^16^ In all, 4,546 children had both phenotypic and genetic data available for analysis, with 2,259 (49.7%) of them being male. A total of 78 children (1.7%) had an ADHD diagnosis (any type) with the majority (70) of them being male.

### Target ADHD Clinical Sample

The target sample consisted of British children with a confirmed *DSM-IV* research diagnosis of ADHD (N = 508) recruited from community child and adolescent mental health and child health clinics. Trained interviewers used the Child and Adolescent Psychiatric Assessment—Parent Version,^23^ a semi-structured research diagnostic interview, to assess psychiatric diagnoses. *DSM-IV* ADHD and symptom domain severity were calculated by summing scores on ADHD items. Pervasiveness of ADHD symptoms (in school) was assessed using the Child Attention-Deficit Hyperactivity Disorder Teacher Telephone Interview^24^ or the Conners Teacher Questionnaire.^25^ Individuals in the ADHD sample had a confirmed lifetime diagnosis of ADHD, but some had remitted symptoms at the time of assessment and are excluded from the analysis of symptom severity.

IQ was assessed using the WISC-IV.^26^ The children in this study were between 5 and 17 years of age (mean = 10.5, SD = 2.8 years), and 443 (87.2%) were male.

DNA samples from children with ADHD were genotyped on the Illumina Human660W-Quad BeadChip, and control participants were genotyped by the Wellcome Trust Case Control Consortium–Phase2 using the Illumina Human 1.2M BeadChip.^27^ Control participants comprised 3,000 individuals born in the United Kingdom during 1 week in 1958 (the 1958 British Birth Cohort) and 3,000 individuals from the UK Blood Services collection. It has previously been shown that it is valid to combine these 2 samples for use as control participants in genetic association studies using UK case samples.^28^ The GWAS case-control analysis was based on 502,702 genotyped SNPs present on both chips after QC. Details on the QC procedure and results on this GWAS have been described previously.^16^

The target sample was selected for this study due to its similarity to the ALSPAC study both in terms of ethnicity and geographic location, as well as its robust diagnostic assessment process. Research participants in the target and discovery samples were recruited from geographically nearby regions (Southwest England and Wales). Therefore, an identity by descent (IBD) analysis was conducted using PLINK^20^ to ensure that there would be no related individuals between the 2 samples. Two individuals in the clinical ADHD sample who showed IBD of 12.5% or more in relation to individuals in the discovery sample were removed from all analyses.

### Statistical Analysis

To identify risk alleles in the ALSPAC discovery sample (quantitative GWAS of ADHD traits), we selected SNPs with a threshold of *p* < 0.5, in line with previous studies.^15,29^

SNPs in linkage equilibrium in the Cardiff clinical sample were identified using PLINK^15^ (–indep-pairwise 200 5 0.2) with a sliding window of 200 SNPs, moving it along the genome 5 SNPs at a time and dropping an SNP when the pairwise estimate of linkage disequilibrium (LD;r^2^) exceeded 0.2.

The SNPs that were present in both the list of risk alleles from ALSPAC and the LD-pruned list from Cardiff (n = 231,488) were used to calculate a polygenic score for each individual in the Cardiff clinical sample, which was a sum of all risk alleles weighted by β coefficient using the PLINK command (–score) with imputation of missing genotypes. The resulting polygenic scores were standardized using z-score transformation and tested for association with ADHD case-control status in the Cardiff sample using logistic regression with sex as a covariate. They were also tested for association with total number of *DSM-IV* ADHD inattentive and hyperactive/impulsive symptoms, using linear regression with sex as a covariate. The amount of variance explained was calculated as the difference of Nagelkerke’s pseudo-R^2^ in the full model as compared to the null model, which included sex but not polygenic score. *p* Values were determined from likelihood ratio tests, which compare the full model to the null model. In addition, conditional analysis was performed with hyperactive/impulsive symptoms included as a covariate in the model for inattentive symptoms and inattentive symptoms included as a covariate in the model for hyperactive/impulsive symptoms to test whether any particular ADHD symptom domain was contributing more to the association. Statistical analyses were performed using Stata Statistical Software Release 13.^30^

## Results

In this study, we used risk alleles from a GWAS of ADHD traits in the general population to calculate polygenic risk scores in a clinical sample of children with ADHD and population cohorts. Table 1 describes the characteristics of individuals used in the discovery sample (ALSPAC GWAS) and the target sample (ADHD clinical sample).

Polygenic risk scores were calculated in the Cardiff cases and controls based on the results of the GWAS of ALSPAC ADHD traits. To test whether increased polygenic score for ADHD traits was associated with case-control status in the clinical study, logistic regression of polygenic risk scores for ADHD traits on 508 individuals with ADHD and 5,081 control participants was used. Results show that population ADHD trait polygenic scores significantly distinguish participants with ADHD from controls (odds ratio [OR] = 1.17 [95% CI = 1.08–1.28], *p* = .0003, pseudo-R^2^ = 0.004).

Table 2 shows the results of association between trait polygenic scores derived from the ALSPAC GWAS and *DSM-IV* ADHD symptom severity and ADHD symptom domain scores in the clinical sample. Increased genetic load for ADHD traits predicted higher ADHD symptom severity in the clinical sample (β = 0.29 [95% CI = 0.04–0.54], *p* = .02, pseudo- R^2^ = 0.011), as well as symptom domain severity. The association with inattentive symptoms (β = 0.17 [95% CI = 0.02–0.33], *p* = .03, pseudo-R^2^ = 0.01) was stronger than that for hyperactive/impulsive symptoms (β = 0.14 [95% CI = −0.01 to 0.29], *p* = .06, pseudo-R^2^ = 0.007), but the direction of effect was as expected.

Table 3 describes the results of the conditional analysis with hyperactive/impulsive symptoms included as a covariate in the model for inattentive symptoms and inattentive symptoms included as a covariate in the model for hyperactive/impulsive symptoms. The magnitude of association of ADHD trait scores with inattentive symptoms was reduced when adjusting for hyperactive/impulsive symptoms, but the direction of effect was still as expected. However, there was no association of ADHD trait scores with hyperactive/impulsive symptoms when adjusting for inattentive symptoms, which suggests that inattentive symptoms contributed more to the association than hyperactive/impulsive symptoms.

## Discussion

The present study is the first to show, using molecular genetics, that the same risk alleles contributing to ADHD trait levels in the general population also confer risk of ADHD diagnosis and increased symptom severity. ADHD trait polygenic risk scores distinguished case individuals with an ADHD diagnosis from general population controls and were associated with a higher number of ADHD symptoms in individuals with an ADHD diagnosis.

Polygenic risk score analysis is well suited for phenotypes such as ADHD. ADHD is a heritable disorder with twin heritability estimates at 76%^31^ and SNP heritability of 24% to 32% according to the latest cross-disorder analysis.^10^ Common genetic variants have been shown to be involved in ADHD at the disorder level.^16^ However, no specific genetic variants have been robustly associated yet in a hypothesis-free GWAS of ADHD.^16,32^ This is likely attributable to the smaller sample sizes compared to those for other psychiatric disorders with similar heritability. For example, 128 independent SNPs spanning 108 distinct loci for schizophrenia were recently identified when sample sizes increased to >80,000 individuals.^33^ For these reasons, research on common genetic variants in ADHD has started focusing on methods that consider common variants from GWAS as an aggregate, namely polygenic risk score analysis. There is evidence that polygenic scores for clinical ADHD can predict case-control status in an independent sample.^13^ In addition, we have previously shown that polygenic risk scores derived from clinical patients with ADHD predict ADHD trait level in the general population.^14^ We now provide evidence using molecular genetics that common gene variation that contributes to ADHD trait levels in the general population is also relevant to risk of clinical disorder and its severity. This is especially pertinent because the amount of variance explained by common variants for behavioral traits in the general population has been considered too low to be relevant to the disorder, and it has been suggested that the heritability of behavioral traits has a large nonadditive genetic influence.^11^

These findings have important implications for ADHD research. First, they provide further support to the notion that ADHD as a disorder lies on the spectrum of normal trait variation. Family and twin studies have already shown that a clinical diagnosis of ADHD in one sibling is associated with increased ADHD trait scores in the unaffected sibling.^34,35^ The present study suggests that, at least at the level of common genetic variants, genetic risk factors for subthreshold ADHD traits present in individuals from the general population and those with the clinical disorder overlap, although the extent of overlap could be limited given small effect sizes. Similar results have been found for physical conditions that are underpinned by continuously distributed risk dimensions (for example, polygenic risk scores for type 2 diabetes are associated with fasting glucose in the general population^36^), strengthening the notion that complex disorders are, in fact, quantitative traits,^37^ and presenting clinicians with the challenge to determine diagnostic and treatment thresholds. Our findings raise questions about the arbitrariness of thresholds used for diagnosis and treatment in psychiatric disorders, especially when children with subthreshold ADHD cannot benefit from access to special educational services. This, together with findings that high levels of ADHD traits in children from the general population carry risk of worse academic performance,^38^ suggests that more research is required to investigate whether recognizing ADHD symptoms, even subthreshold, at an early stage and offering appropriate support can reduce the risk of educational underachievement and problematic behaviors.

More importantly, these results are relevant to the debate of whether the genetic architecture of behavioral traits differs from that of other quantitative traits. A recent molecular genetic study of twins reported a discrepancy of heritability estimates for behavioral traits between twin estimates and GCTA in the same sample of children at age 12 years.^11^ No genetic influence for behavioral traits could be detected using GCTA at age 12 years, whereas heritability estimates from the twin design were substantial, and others report significant GCTA estimates for social communication traits in older age groups.^4^ Both GCTA and the twin design showed substantial heritability for cognitive and anthropometric traits in the same sample.^11^ This has led to speculation that additive genetic influences might not be as relevant for behavioral traits as they are for other quantitative traits or that the phenotype assessment based on questionnaire ratings rather than standardized tests influences heritability estimates. GCTA was not appropriate for ADHD traits in ALSPAC, given that the variable is highly skewed and difficult to transform appropriately. Using polygenic risk score analysis, we were able to counter these suggestions by showing that additive genetic variance was relevant for ADHD even when it was based on ADHD trait scores measured in children from the general population. The discrepancy of our results with recent GCTA reports could be due to a number of factors. First of all, we had a larger sample size of 4,546 children available as compared to 2,500 unrelated individuals in the previous report.^11^ ADHD traits in children were assessed with the DAWBA, which is a semi-structured diagnostic interview. The use of more clinically rigorous instruments for assessing symptoms can influence results in this type of analysis. Finally, the ALSPAC sample is very homogeneous in terms of ancestry, as all participants are from the Bristol area in the United Kingdom.

Adjustment of the associations with ADHD symptom domains suggests that the association of ADHD trait scores with the number of ADHD symptoms is driven by inattentive symptoms rather than hyperactive/impulsive symptoms. This could indicate that the genetic component of inattentive symptoms is stronger than that for hyperactive/impulsive symptoms. An alternative explanation could be that genetic influences on hyperactive/impulsive symptoms that do not overlap with genetic influences on inattentive symptoms may contribute more to ADHD traits in the general population. Although some twin studies have found distinct genetic influences on symptom domains, the evidence on the whole is not compelling.^39^ Indeed, ADHD subtype diagnoses have been removed in the *DSM-5*, and they serve only as descriptive specifiers. Despite the lack of distinct genetic influences for ADHD symptom domains, inattentive symptoms are developmentally more persistent than hyperactive/impulsive symptoms.^40^

Our study benefits from a large discovery population sample that is not only ancestrally similar to the well-characterized clinical sample used but also geographically adjacent (while any related individuals between the samples were removed from the analysis). Both samples are homogeneous in terms of their ancestry and also diagnostic assessment. Although each study used different diagnostic instruments, the assessment within each sample was standardized across all individuals.

A limitation of the study is that there is no phenotypic information available on the controls in the case-control ADHD study. This means that there could be individuals with ADHD in the control sample that we were unable to identify. However, this would only reduce our power to distinguish between individuals with ADHD and controls. In a future study, it would be interesting to test whether polygenic scores were associated with ADHD trait levels in individuals from another general population cohort. Unfortunately this was not possible in this study because of the lack of ADHD trait scores in the control sample.

As a longitudinal study, the ALSPAC cohort suffers from attrition that could be associated with behavioral problems, such as ADHD. In this case, the predictive power of the polygenic scores would be reduced and the associations with ADHD would be weaker. However, the prevalence of ADHD in the ALSPAC sample was 3.1% for boys and 0.3% for girls, which is very similar to the UK prevalence for ADHD during childhood (2.7% for boys and 0.4% for girls).^41^ Multiple imputation methods for missing data have been applied to ALSPAC ADHD data previously but did not have an effect on the association patterns.^42^

Another potential issue that should be discussed is that because the target sample consists of children who already have a diagnosis of ADHD, a significant proportion of them were likely to be on medication that reduces ADHD symptom severity. Although this would have an impact on the power to detect an association with the number of ADHD symptoms, it would not have an impact on our ability to distinguish between individuals with ADHD and controls, because children with ADHD had a lifetime diagnosis based on symptoms present before initiation of medication.

In summary, polygenic scores for ADHD trait levels in the general population are associated with an ADHD diagnosis and with symptom severity for those with the disorder. This study highlights the importance of common genetic variants for ADHD traits and the dimensionality of the ADHD phenotype. It also counters suggestions that the genetic architecture of behavioral traits is different from that of other complex traits.

**Clinical Guidance**

• Genetic risk factors for subthreshold ADHD traits present in individuals from the general population and those with the clinical disorder overlap.
• There is further support to the notion that ADHD as a disorder lies on the spectrum of normal trait variation.
• Additive genetic variance is relevant to the genetic architecture of behavioral traits not unlike other quantitative traits.
• More research is required to investigate whether recognizing ADHD symptoms, even subthreshold symptoms, at an early stage and offering appropriate support could reduce the risk of educational underachievement and problematic behaviors.

## Figures and Tables

**Table 1:** Descriptive Statistics for Avon Longitudinal Study of Parents and Children (ALSPAC) Genome-Wide Association Study (GWAS) and Cases From Attention-Deficit/Hyperactivity Disorder (ADHD) Clinical Sample

	ALSPAC GWAS	ADHD Clinical Sample Cases
n	4,546	508
% Male	49.7	87.2
Age in months, mean (SD)	91.8 (1.7)	126 (33.6)
ADHD traits/clinical symptoms, n (SD) [range]	4.6 (6.4) [0-36]	14.7 (2.8) [0-18]
Full Scale IQ, mean (SD)	106.6 (15.6)	87.2 (11.2)

**Table 2:** Linear Regression of Polygenic Scores for Attention-Deficit/Hyperactivity Disorder (ADHD) Traits on the Total Number of ADHD Symptoms and Symptom Domains in Children With an ADHD *DSM-IV* Diagnosis

Outcome	β Coefficient (95% CI)	*p* Value	R^2^	n
Total no. of *DSM-IV* ADHD symptoms	0.29 (0.04 to 0.54)	.02	0.011	484
Total no. of *DSM-IV* inattentive symptoms	0.17 (0.02 to 0.33)	.03	0.01	493
Total no. of *DSM-IV* hyperactive/impulsive symptoms	0.14 (−0.01 to 0.29)	.06	0.007	497

Note: n differs from case-control analysis because of individuals with remitted symptoms at the time of assessment who are excluded from the analysis of symptom severity.

**Table 3:** Conditional Linear Regression of Polygenic Scores for Attention-Deficit/Hyperactivity Disorder (ADHD) Traits on ADHD Symptom Domains in Children With an ADHD *DSM-IV* Diagnosis

Outcome	β Coefficient (95% CI)	*p* Value	R^2^	n
Total no. of *DSM-IV* inattentive symptoms adjusting for hyperactive symptoms	0.13 (−0.02 to 0.27)	.09	0.002	493
Total no. of *DSM-IV* hyperactive/impulsive symptoms adjusting for inattentive symptoms	0.08 (−0.06 to 0.22)	.26	0.005	493
